# Unlocking the neural mechanisms of consumer loan evaluations: an fNIRS and ML-based consumer neuroscience study

**DOI:** 10.3389/fnhum.2024.1286918

**Published:** 2024-02-05

**Authors:** Tuna Çakar, Semen Son-Turan, Yener Girişken, Alperen Sayar, Seyit Ertuğrul, Gözde Filiz, Esin Tuna

**Affiliations:** ^1^Department of Computer Engineering, MEF University, Istanbul, Türkiye; ^2^Department of Business Administration, MEF University, Maslak, Türkiye; ^3^Faculty of Economics and Administrative Sciences, Final International University, Istanbul, Türkiye; ^4^Informatics Technologies Master Program, MEF University, Istanbul, Türkiye; ^5^Computer Science and Engineering Ph.D. Program, MEF University, Istanbul, Türkiye; ^6^Department of Psychology, MEF University, Istanbul, Türkiye

**Keywords:** financial decision-making, functional near-infrared spectroscopy (fNIRS), neural activation patterns, prefrontal cortex (PFC), consumer neuroscience, credit decision prediction, machine learning algorithms, neurofinance

## Abstract

**Introduction:**

This study conducts a comprehensive exploration of the neurocognitive processes underlying consumer credit decision-making using cutting-edge techniques from neuroscience and machine learning (ML). Employing functional Near-Infrared Spectroscopy (fNIRS), the research examines the hemodynamic responses of participants while evaluating diverse credit offers.

**Methods:**

The experimental phase of this study investigates the hemodynamic responses collected from 39 healthy participants with respect to different loan offers. This study integrates fNIRS data with advanced ML algorithms, specifically Extreme Gradient Boosting, CatBoost, Extra Tree Classifier, and Light Gradient Boosted Machine, to predict participants’ credit decisions based on prefrontal cortex (PFC) activation patterns.

**Results:**

Findings reveal distinctive PFC regions correlating with credit behaviors, including the dorsolateral prefrontal cortex (dlPFC) associated with strategic decision-making, the orbitofrontal cortex (OFC) linked to emotional valuations, and the ventromedial prefrontal cortex (vmPFC) reflecting brand integration and reward processing. Notably, the right dorsomedial prefrontal cortex (dmPFC) and the right vmPFC contribute to positive credit preferences.

**Discussion:**

This interdisciplinary approach bridges neuroscience, machine learning and finance, offering unprecedented insights into the neural mechanisms guiding financial choices regarding different loan offers. The study’s predictive model holds promise for refining financial services and illuminating human financial behavior within the burgeoning field of neurofinance. The work exemplifies the potential of interdisciplinary research to enhance our understanding of human financial decision-making.

## 1 Introduction

Since Plato, followed by his pupil Aristotle, humans have been characterized as rational animals (Keil and Kreft, 2019). This assumption was so central to the way humans conceived of themselves that it served as the foundation for entire legal and economic systems (Blasi and Jost, 2006). Normative theories of decision-making, such as the St. Petersburg paradox, introduced by the 18th-century mathematician Daniel Bernoulli, prescribes optimal approaches to decision-making (Bernoulli, 1954). Bernoulli’s explanation was based primarily on the distinction between the objective value (i.e., the expected utility) and the subjective value (i.e., the expected benefit) of the potential monetary gains. The lack of human-related materials has led to the aforementioned distinction in order to fully and rationally explain such a paradox. In the mid-20th century, mathematician John von Neumann and economist Oskar Morgenstern established the underlying assumptions of Expected Utility Theory (Von Neumann and Morgenstern, 1944) and asserted that if certain conditions were met, individuals’ financial decisions could be modeled through a utility function (Peasgood et al., 2014). However, although these theoretical frameworks are valuable, they are limited in explaining how humans make decisions in both hypothetical and real-life scenarios. Nearly two millennia later, following the emergence of behavioral and cognitive sciences, the question of human rationality started to be a subject of debate among scholars. With the popularization of prospect theory (Kahneman and Tversky, 1979), which challenged the premise of fully rational behavior by offering several examples of human biases such as framing, subjective reference points, loss aversion, and the isolation effect, research on the mechanisms which prevent humans to act rationally grew. Empirical studies on heuristics, cognitive biases, and situations that can induce irrational behavior also increased rapidly (De Martino et al., 2006), and the interest of the scientific community in the neural basis of financial decision-making processes followed suit.

### 1.1 Behavioral finance

Behavioral finance integrates insights from various fields of research, drawing upon the works of several eminent scholars, including Nobel laureates. Among them, Herbert Simon introduced the concept of bounded rationality to explain how humans’ cognitive limitations influence their decision-making processes, challenging conventional economic models that assume fully rational agents (Simon, 1957). Richard Thaler, another Nobel laureate, started a significant departure from the prevailing prescriptive economic framework, drawing on the insights of early cognitive psychologists such as Kahneman and Tversky, and co-developed the concept of nudging (Kahneman and Tversky, 1979; Sunstein and Thaler, 2008). Over time, a growing body of research has documented deviations from rational decision-making processes, demonstrating that individuals consistently and predictably deviate from the assumptions of traditional finance models. In addition to these, Shiller (1981) provided compelling evidence for market inefficiencies, challenging the assumption of investor rationality that underpins the efficient markets hypothesis. Shiller’s (1981) work on capital markets played a pivotal role in garnering the attention of financial researchers to behavioral finance.

The works of Fama (1970, 1990) Nobel laureate Harry Markowitz laid the groundwork for what is commonly referred to as “traditional” finance. Markowitz’s (1952) modern portfolio theory (MPT) assumes that rational investors base their decisions on two key criteria: expected reward and variance. Behavioral finance gained traction among academics due to the identification of various market inefficiencies and the limitations of prevailing traditional finance models, including MPT, to account for these anomalies. Advocates of behavioral finance extensively employed cognitive psychology theories to explain suboptimal investor behavior or the decision-making biases of sentiment-driven traders (Black, 1986; De Long et al., 1989). The motivation to gain a deeper understanding of financial decision-making processes has motivated researchers to adopt novel methodologies and frameworks, primarily focusing on neuroscientific applications, and more notably neurofinance. Initial efforts in neurofinance have focused on elucidating the neural mechanisms underlying humans’ daily financial behavior, utilizing brain imaging techniques. Neurofinance has emerged as a distinct field driven by advancements in brain imaging technologies and empirical evidence drawn from neuroscience, finance, and experimental finance. Neurofinance has significantly enhanced our understanding of neural processes involved in financial decision-making processes, including credit decisions and investment decisions.

### 1.2 Financial decision-making

Financial decision-making results from complex neural processes involving, among other factors, the ongoing evaluation of relevant statistical data, the balancing of emotional factors, and the calculation of essential value indicators crucial for modern economic thought (Bossaerts, 2009). In financial decision-making, several factors, including risk and uncertainty, need to be evaluated. The purpose of these assessments is to obtain rewards, in other words, a favorable outcome or benefit anticipated from a specific action or decision. For example, financial risk assessment involves balancing potential gains against potential losses (Knutson and Bossaerts, 2007). As reward processing is influenced by homeostatic and psychological needs and goals, it interacts with emotional processing. Therefore, the influence of both incidental emotions and outcome anticipation on financial decision-making seems to be an essential aspect of the decision-making process (Sander and Nummenmaa, 2021; Sandberg et al., 2022).

#### 1.2.1 Impact of reward system on financial decisions

From an evolutionary perspective, the reward concept is categorized into two primary categories. Primary rewards encompass essential survival needs, such as food or sex. Secondary rewards derive their value from their association with primary rewards, such as monetary gains (Miendlarzewska et al., 2019). In other words, for centuries, primary rewards could be obtained with secondary rewards such as money. While the limbic system focuses on the quantity of primary rewards, the prefrontal cortex is responsible for making strategic decisions regarding secondary rewards to obtain these primary rewards. When making such strategic decisions, it is crucial to evaluate the potential gains and losses and ensure that the value gained outweighs the value given. There is an internal motivation to make the right decision by evaluating current conditions while drawing upon past experiences (Beck et al., 2010; Rushworth et al., 2011; Sescousse et al., 2013).

The neural correlates of financial decision-making are underpinned by the integration of information from diverse mental domains and by the operation of intricate neurobiological processes within the human brain (Erkut et al., 2018). Investigating neural activity in prefrontal cortex areas during financial decisions can therefore yield novel insights into this complex cognitive domain. Dopaminergic systems constitute the brain’s reward circuitry, responsible for encoding and evaluating reward-related cues. Financial stimuli are perceived as a form of monetary incentive, capable of activating the reward system and influencing decision-making (Walter et al., 2005; Burgdorf and Panksepp, 2006; Bermejo et al., 2011; Lewis et al., 2021). Dopamine receptors, in particular, modulate risk-seeking and risk-averse tendencies, affecting the influence of risk–reward trade-offs (Kuhnen and Chiao, 2009; Soutschek et al., 2023). Just as purchasing decisions are characterized as a form of problem-solving behavior (Kotler, 1997), decisions related to credit offers can be conceptualized as a decision-making process involving the identification of the credit offer, the appraisal of the credit offer terms, and the formulation of a decision.

The purchasing process necessitates the consideration of a multitude of factors, including price, size, and other features, with the aim of optimizing value and contentment (Engel et al., 1995). Decisions regarding credit offers are potentially influenced by various internal factors, such as the principal amount, interest rate, and repayment period, all of which comprise both emotional and rational considerations, as well as by external factors. For instance, provided that monthly payments remain within the borrower’s affordability range, there is a propensity for price escalation as consumers exhibit a tendency to enter into extended payment obligations (Shin et al., 2020). An individual’s decision to take on a loan appears to be influenced by a combination of factors, including their personal history, their current economic situation, and episodic emotions such as perceived risk and uncertainty about the future (Lerner et al., 2015). Before this decision becomes conscious to the individual, it seems that a significant activation in the prefrontal regions is necessary to facilitate optimal goal-directed decision-making. The prefrontal cortex is associated with various higher-order cognitive functions, including problem-solving, decision-making under uncertainty, strategic thinking, working memory, and cognitive control (Funahashi, 2017).

#### 1.2.2 The role of the prefrontal cortex on financial decisions

A comparative study of products from national and private brands demonstrated that products perceived as being of higher quality elicit relatively greater left frontal activation during the pre-decision phase (Ravaja et al., 2013; Bosshard et al., 2016). In essence, these findings suggest that the neural activation of the left prefrontal cortex may serve as an indicator for positive expectations of gain. This left frontal activation might indicate an integrated representation of neural processes throughout the prefrontal cortex (PFC). However, few experimental studies have revealed differential oxygenation levels in the right dorsolateral prefrontal cortex during choices between labeled and unlabeled products, as well as between strong and weak brands (Tusche et al., 2010; Kapoor et al., 2023). Delving into the underlying mechanisms of this process is essential. Empirical evidence indicates that the activity of the bilateral vmPFC intensifies during preference judgments (“Which one do you like better?”) compared to visual discrimination tasks (“Which drink is in the bottle?”).

Moreover, three other brain areas exhibited significant differences in activation between visual discrimination tasks and preference judgments: the anterior cingulate gyrus, the left insular cortex, and the right posterior parietal lobule. During visual discrimination tasks, a significant positive correlation was observed between activation levels in the anterior cingulate gyrus and the number of discrimination errors (Paulus and Frank, 2003). An investigation of neural activity in the PFC revealed that when individuals made purchase decisions based on price, oxygenation modulation in the right anterior polar cortex significantly influenced purchase decisions (Mitsuda et al., 2012; Çakır et al., 2018). Specifically, the left dlPFC is implicated in decision-making when exposed to rising prices in addition to the vmPFC activation which is associated with cash, stock holdings, and trade-offs in financial decision-making during economic bubbles (Ogawa et al., 2014).

In contrast, when faced with an unfair offer, especially one in which fairness and self-interest clash, functional connectivity between the posterior vmPFC and the right dlPFC intensifies. During the evaluation of unfair offers, the right dlPFC contributes to the ability to make normatively aligned decisions even when they involve personal sacrifices. Conversely, the deactivation of the posterior vmPFC impairs the ability to make normatively aligned decisions despite personal costs. The orbitofrontal cortex (OFC), located within the prefrontal cortex, has been shown to be active in various studies examining business strategies, purchase choices, product selection, risk evaluation, financial investment decisions, and price assessments. Neural activity of the OFC is associated with the anticipation of rewards. When comparing high-profit margins to low-profit margins, the OFC exhibited reduced activation or no activation in response to low-profit margins. Like the OFC, the right dlPFC also exhibited reduced activation when low-profit margins were presented. Moreover, activation in the right OFC intensifies when presented with images of little desirable food, suggesting that this region plays a crucial role in processing negative emotional responses. A multitude of factors influence purchasing decisions, including musical cues, monetary considerations, and social evaluations. Additionally, perceptual assessments of desirable and undesirable product features play a significant role in shaping purchasing decisions (Kumagai, 2012).

An empirical study investigated the brain response of consumers to various commercial communication strategies at the point of sale. The neuroimaging data revealed a significant effect on the perception of marketing strategies in two specific brain regions: the OFC and the dorsolateral prefrontal cortex (dlPFC) (Krampe et al., 2018a). Furthermore, numerous empirical studies examining financial decision-making processes have yielded insights on a range of topics such as the influence of ambiguity, the distinction between risk and ambiguity (Huettel et al., 2006; Coutlee et al., 2016), the neural representation of ambiguity in the fronto-cortical regions (Payzan-LeNestour et al., 2013), and the evaluation and comparison of subjective values in the OFC (Levy et al., 2010; Padoa-Schioppa and Cai, 2011).

Risk-taking behaviors possess an adaptive value in acquiring potential benefits, both tangible and intangible. However, the gains and losses should be precisely assessed, particularly in uncertain situations (Hsu et al., 2005). In terms of financial decisions, the evaluation of risk fundamentally hinges on the ability to control impulsive tendencies, which can be modulated by the magnitude heuristic. This heuristic can be explained by people assigning less value to options with low potential gains compared to options with high potential gains. Therefore, it is necessary to suppress impulsivity to anticipate scenarios where the potential losses are significant or the option with an excessively high-risk profile is chosen. The decrease in the neural activity of the right dlPFC diminishes the influence of this heuristic, impeding the ability to control impulsive reactions (Ballard et al., 2018). On the other hand, increased activity in the left dlPFC and decreased activity in the right dlPFC are associated with risk-taking in the context of loss, especially when protection is a concern (Huang et al., 2017). Elevated activity in the right dlPFC also contributes to a higher propensity for risk-taking (Yang et al., 2017).

### 1.3 Neuromarketing and strategies for studying consumer behavior

One of the central goals of applied neurobiology is to unravel the cognitive and neural mechanisms underpinning purchase decisions (Meyerding and Mehlhose, 2020). Diverse attributes, such as price, brand, and product features, can influence individuals’ preferences, and the individuals themselves are also a crucial factor within this context (Venkatraman et al., 2012). Marketing researchers and experts have traditionally employed conventional marketing methodologies including field studies, surveys, focus groups, and in-depth interviews, to uncover individuals’ preferences and the factors that shape those preferences (Ariely and Berns, 2010). However, the past two decades have seen the emergence of the field of neuromarketing, which leverages neuroscientific techniques to gain a deeper understanding of individual decision-making processes, emotional responses, memory formation, value systems, and attitudinal dispositions in response to marketing interventions (Ariely and Berns, 2010; Plassmann et al., 2012, 2015; Çakar et al., 2021).

Neuroscientific methods such as functional magnetic resonance imaging (fMRI) and electroencephalography (EEG) are utilized in applied neurobiology research to monitor consumer behavior and decision-making. However, due to the challenges in real-world implementation of these methods, fNIRS has gained prominence in recent years (Krampe et al., 2018b; Lee et al., 2018). This optical neuroimaging method strives to elucidate human brain processes by assessing cortical hemodynamic responses. fNIRS also has extensive applications in both medical and neuroscience research (Ferrari and Quaresima, 2012; Huo et al., 2021). Numerous studies have demonstrated that a wide range of phenomena, including personal preferences, preference intensity, risk-taking behavior, and task performance, can be detected using fNIRS (Kumagai, 2012; Holper et al., 2014; Kim et al., 2016). Since it is user-friendly and yields accurate results, machine learning researchers have also begun using this technique (Ferrari and Quaresima, 2012). Once deep learning approaches are employed in investigations that use fNIRS, many of the challenges that we currently encounter, such as time-consuming data preparation and restricted sample sizes, will be reduced while still obtaining comparable or superior classification accuracy (Eastmond et al., 2022).

The synergy between fNIRS and machine learning algorithms has shown promising results in predicting various cognitive states and behaviors. An overall accuracy rate exceeding 75% was achieved when investigating whether referring to a product as expensive or inexpensive could influence its perceived value-for-money (Misawa et al., 2014). In mental imagery, an accuracy of over 96% was achieved (Shibu et al., 2023). Moreover, these machine learning models can differentiate between mind wandering and task-related episodes with an accuracy of 73.2% (Liu et al., 2021). In finger-tapping task classification, an accuracy of 99.52% was achieved (Sommer et al., 2021), and in pain perception task, an accuracy of 94.17% was attained (Fernandez Rojas et al., 2019). These studies provide evidence to support the notion that consumer behavior may be predicted by combining fNIRS and machine learning algorithms.

### 1.4 Current research framework

The present study endeavors to develop a classification model using machine learning algorithms trained on participants’ hemodynamic response data to predict their acceptance or rejection of bank loan offers. This study investigates the association between credit decisions and brain activity patterns in the prefrontal cortex. The main research question is formulated as such: “Is there a difference in neural activation between positive and negative credit decisions?” Hereby, we used fNIRS to investigate the neural mechanisms underlying decision-making toward credit offers and their impact on the PFC. Thus, we hypothesized an active involvement of PFC supporting reward anticipation and risk evaluation, including vmPFC, dmPFC, and dlPFC, that will end up with accepting or rejecting a credit offer.

The originality of this research stems from its pioneering combination of the fNIRS technology and advanced machine learning algorithms to investigate the neural mechanisms underpinning individual credit decision-making processes. By identifying unique patterns of neural activation within specific prefrontal cortex regions, this study provides unprecedented insights into the cognitive and emotional processes that shape financial preferences. Moreover, this research contributes to the burgeoning fields of consumer neuroscience and neurofinance by proposing a predictive model that not only enhances our understanding of individual credit decisions but also holds the potential to inform the development of personalized financial services and strategies.

## 2 Materials and methods

### 2.1 Participants

A total of 39 participants (21 female) residing in Türkiye were recruited to take part in this study. The age of the participants ranged from 18 to 46 years. The mean age and standard deviation of participants before preprocessing were 32.75 (SD = 5.05) for females and 34.3 (SD = 5.78) for males, the result of the statistical test indicated no significant age difference based on gender [*t*(34) = −0.84, *p* = 0.404]. Moreover, the age distribution of the participants with respect to their educational background also showed no significant difference with respect to age [*t*(32) = −0.56, *p* = 0.58]. The mean age and standard deviation of participants before preprocessing were 32.89 (SD = 4.65) for high school graduates and 33.94 (SD = 6.27) for Bachelor’s degree holders, the result of the statistical test indicated no significant age difference based on gender [*t*(34) = −0.84, *p* = 0.404].

The participants were selected from the consumer database of a marketing research company via a stratified sampling method across gender. All participants were right-handed as measured by the Edinburgh Handedness Inventory (Oldfield, 1971). None of the participants had a history of psychiatric disorders. Participants were paid a monetary incentive for their participation. The data of 3 participants were excluded due to excessive movements and artifacts. This study was approved by the research ethics committee of MEF University (47749665-050.01.04/275). Written informed consent was obtained from the participants prior to the experiment.

### 2.2 Optical brain imaging system

The fNIRS is a non-invasive technique to monitor cerebral hemodynamics, detecting variations in blood oxygenation caused by neural activity. This technique qualifies as an effective tool for neuroimaging applications outside traditional laboratory settings. Essentially, fNIRS accurately tracks cerebral oxygenation and blood flow dynamics (Bunce et al., 2006). In our study, we utilized a continuous-wave fNIRS system designed and provided by fNIR Devices LLC (Potomac, MD, USA).^1^ This system includes a flexible headpiece (sensor pad) accommodating 4 light sources and 10 detectors, enabling the measurement of oxygenation levels in the prefrontal cortex via 16 optodes. It also comprises a control unit to manage hardware operations and a computer with COBI Studio software to facilitate data collection (Ayaz et al., 2011). COBI Studio Software was synchronized with E-Prime Software (v2.0) which was used for stimuli presentation with the use of markers sent via parallel port, and fNIRSoft was used for data preprocessing and the first stages of the analyses (Ayaz, 2010).

**FIGURE 1:**
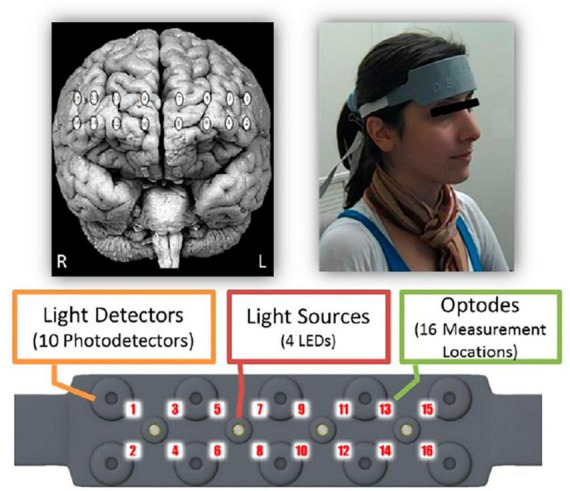
Spatial distribution of monitored cortical regions using functional near-infrared spectroscopy (fNIRS). Reproduced with permission from Ayaz (2010).

The sensor features a source–detector distance of 2.5 cm, enabling a penetration depth into the brain of approximately 1.25 cm. This configuration enables the system to track relative shifts in oxyhemoglobin (HbO), i.e., oxygen-laden hemoglobin, and deoxyhemoglobin (HbR), i.e., hemoglobin that has released its oxygen load. These shifts in HbO and HbR concentrations, along with the total hemoglobin (HbT), i.e., the sum of HbO and HbR, and the oxygen saturation (Oxy), i.e., the percentage of oxygenated hemoglobin in the blood, are monitored with a temporal resolution of 2 Hz. Neural activity assessment hinges on these oxygenation changes, as variations in the brain blood flow are intricately linked to functional brain activities through a process known as neurovascular coupling (Obrig et al., 2000). The related optodes to this coupling were positioned on the scalp at locations corresponding to Brodmann areas 9, 10, 44, and 45, as depicted in Figure 1 (Ayaz et al., 2012).

### 2.3 Experimental procedure

The task comprised 35 scenarios in which participants were presented an offer and asked to make credit decisions based on the current market. In each block, the participants had 7 s to view a picture of the offer, and 5 s to respond to the offer with a binary decision (“Yes” or “No”) according to whether they would prefer to apply or not to apply; finally, an 8-s fixation screen completed the block, so that each block lasted for 20 s. The total duration of the experiment was approximately 12 min. E-Prime software (V2.0) was used for the presentation of the experiment stimulus. The participants were told that they should press a button to indicate whether they would or would not apply to the offer displayed on the screen, based on the details provided. The keys participants needed to press to indicate their preference were randomly switched in each block to avoid lateralization biases.

The presented offers contain the details of a loan offer which includes a term of months (24 months for instance), indicating the duration over which the loan must be repaid. The interest rate applied to the loan is also presented (such as 2.39%), which affects the total cost of borrowing. The principal amount of the loan, or the total amount borrowed, is 30,000 units. The borrower is required to make monthly installments of 1,746.33 units to repay the loan. Over the course of the loan term, the total amount to be repaid, which includes both the principal and the accrued interest, sums up to 41,911.88 units. These components are key in understanding the financial obligations of the loan, including how much is borrowed, the cost of borrowing, and the repayment schedule. Yet, these components are presented in a single screen for each offer during the experiment.

### 2.4 Data preprocessing

The preprocessing of the dataset was conducted to reflect the temporal interplay between the presentation of stimuli and the resultant changes in tissue oxygenation. We utilized fNIRS data gathered from 16 optodes operating at dual wavelengths, and applied a 20th-order, 0.1 Hz low-pass filter. This filtering, as advised in by Ayaz et al. (2012), was critical for minimizing high-frequency disturbances stemming from respiratory and cardiac activities. Any channels that exhibited saturation–a state where the detector’s light intensity surpassed the analog-to-digital conversion threshold–were set to null values. We also implemented a robust approach to identify and rectify motion-related artifacts using a sliding window motion artifact filter, as described by Ayaz et al. (2011). The data was segmented into rest and task-related epochs, based on time synchronization markers embedded within the continuous data stream. For each optode and during each task block, such as loan offers and decision-making, changes in blood oxygenation (ΔHbO2 and ΔHbR) were calculated. This was done using the modified Beer-Lambert Law and compared against a baseline oxygenation level established at the onset of each trial (Ayaz, 2010). In the subsequent preprocessing stage, the mean activity during the rest periods preceding each task was subtracted from the activity during each pairing phase. This process helped in highlighting the stimulus-driven effects. We then computed the average values for each phase of the pairing, setting the stage for further comparative analyses. The recorded oxygenation signals, derived by averaging the six-second trials and calculated as ΔHbO2 minus ΔHbR, were then systematically categorized based on the participants’ decisions. These categorized signals were later employed as target classes in our ensuing data analysis endeavors.

Our dataset comprised 1,365 observations, which were obtained from 39 participants each responding to 35 different stimuli, and covered 66 attributes. These attributes comprise the “Participant” identifier and the “Response Code,” along with the values of HbO, HbR, HbT, and Oxy, measured from 16 separate optodes, amounting to 64 metrics in total. The “Participant” column serves as an index for the train-test split process, while the “Response Code” reflects the participants’ binary responses to the loan offers (classified as “Yes/Positive” or “No/Negative”). In our predictive model, the 64 metrics derived from the optodes are used as features, while the “Response Code” serves as the dependent variable we aim to predict. This distinction ensures that our model focuses on predicting the outcomes based on objective neuroimaging data, rather than participant identifiers. We screened the “Response Code” for any invalid entries and excluded them and then excluded rows containing more than 28 null columns during preprocessing. After these steps, the data from three individuals, as well as specific observations for some participants, were removed in the preprocessing phase. After the removal of 3 participants from the dataset, the mean age and the standard deviation for the remaining 36 participants were 33.36 (SD = 5.09) for females and 34.32 (SD = 5.94) for males no significant difference across gender was observed [*t*(31) = −0.48, *p* = 0.63]. Similarly, the mean age and standard deviation for the 36 participants were 33.0 (SD = 4.89) for high school graduates and 34.4 (SD = 6.20) for bachelor’s degree holders; no significant difference across educational background was observed [*t*(29) = −0.70, *p* = 0.49].

We identified outliers within the data of each participant using a z-score methodology. This approach involved comparing each value against the mean and standard deviation specific to that dataset. Any value exceeding a z-score threshold of 4 was adjusted to its nearest boundary value, and this adjustment was uniquely applied for each “Response Code” category. Following this, we employed an Iterative Imputer method for filling in missing data. This technique uses a form of regression modeling that iteratively applies to each variable. It leverages the inter-variable relationships to estimate the missing data points, as explained in Azur et al. (2011). To achieve variance normalization across the different measurement columns, we implemented the standard scaler algorithm. This algorithm normalizes the data range for each individual by centering each feature around the mean and scaling it to unit variance. It effectively standardizes each feature to have a mean of zero and a standard deviation of one, as explained by Pedregosa et al. (2011).

We adopted a stratified hold-out approach, allocating 987 observations for training and 195 for testing, derived from a random selection of 8 participants. The hold-out method is a prevalent validation strategy in the realm of machine learning. It entails reserving a portion of the data as a separate subset, which is not used during the model’s training phase but instead utilized to evaluate the model’s performance. By choosing participants for this process, we ensured that the data used in evaluation was completely independent from the training data. This approach is crucial for a more precise evaluation of the model’s ability to generalize, as highlighted by Hastie et al. (2009).

### 2.5 Handling imbalanced data

The distribution of the “Response Code” within our training dataset is presented in Table 1. Recognizing the challenge posed by the imbalanced nature of the processable data, we employed both oversampling and undersampling techniques to address the issue (Leevy et al., 2018). For oversampling, we utilized the KMeans SMOTE algorithm, setting a cluster balance threshold at 0.3. The KMeans SMOTE algorithm is a synthetic minority oversampling technique that utilizes KMeans clustering to generate synthetic samples in a more targeted manner (Chawla et al., 2002). As for undersampling, the Random Under Sampler algorithm was implemented. This algorithm randomly selects a subset of data for the majority class, reducing it to match the size of the minority class, thereby balancing the dataset (Chen et al., 2013). In addition to these approaches, to observe the differences between the methods, we also developed models without the application of either technique.

**TABLE 1:** Distribution of the response code in the training dataset.

	Count of response code
0 (negative/no)	613
1 (positive/yes)	374

### 2.6 Model development

In the model development phase of our study, we utilized Python 3.10.12, enhanced with essential libraries such as SHAP 0.43.0, catboost 1.2.2, sklearn 1.2.2, imblearn 0.10.1, and xgboost 2.0.2, for data processing, algorithmic exploration, and model optimization. During the model development, we thoroughly examined the impact of various machine learning algorithms on our preprocessed dataset. This investigation encompassed several key steps, including model selection, feature selection, fine-tuning of hyperparameters, training, and a detailed evaluation, allowing for a comprehensive comparison of the performance of different algorithms.

A large range of algorithms was applied to our dataset, namely Logistic Regression (Pampel, 2021), Random Forest (Breiman, 2001), Decision Tree (Dietterich, 2000), AdaBoost (Bühlmann and Hothorn, 2007), XGBoost (Chen and Guestrin, 2016), Extra Tree Classifier (Geurts et al., 2006), CatBoost (Dorogush et al., 2018), and LightGBM (Lin et al., 2022). These algorithms, each with unique characteristics and strengths, are widely recognized and employed in predictive modeling research, particularly within the field of neuroscience. This has been documented, for instance, in the comprehensive review by Glaser et al. (2019), which focuses on the applications of machine learning in neuroscience. The specific methodologies we used, detailed in Table 2, highlight the optimization strategies for each algorithm, guiding the selection of the most suitable models for different types of analytical tasks.

**TABLE 2:** Machine learning algorithms used for model development.

Machine learning algorithm	Strengths	Weaknesses
Logistic Regression	– Simple, interpretable linear model. – Fast to train and predict. – Effective for binary classification problems. – Outputs well-calibrated predicted probabilities.	– Assumes linear relationship between predictors and target. – Can be outperformed by complex models on non-linear data. – Sensitive to irrelevant features and outliers. – Not suitable for complex relationships without feature engineering.
Random Forest	– Robust against overfitting due to ensemble approach. – Handles high-dimensional data well. – Suitable for both classification and regression tasks. – Provides feature importance ranking. – Resistant to noise and outliers.	– May require careful tuning of hyperparameters. – Can be computationally intensive for large datasets. – Interpretability of individual trees might be challenging. – Prone to bias in favor of dominant classes. – Difficult to visualize complex interactions.
Decision Tree	– Easily visualized and interpreted. – Can handle both numerical and categorical data. – Does not require data normalization. – Can model non-linear relationships.	– Prone to overfitting, especially with deep trees. – Can be unstable with slight data changes. – Biased with imbalanced datasets.
AdaBoost	– Boosting technique improves weak learners. – Less prone to overfitting. – Aggregates results for improved accuracy. – Adapts quickly to changes in the data.	– Sensitive to noisy data and outliers. – Requires careful tuning of hyperparameters.
XGBoost	– Powerful ensemble method with high predictive accuracy. – Handles missing data effectively. – Regularization and pruning prevent overfitting. – Supports various evaluation criteria. – Handles imbalanced classes through weighted sampling.	– Prone to overfitting if hyperparameters are not properly tuned. – Requires careful selection of learning rate and tree-specific parameters. – Can be computationally intensive. – Black-box nature makes interpretation challenging. – Potential for biased predictions if not balanced properly.
Extra Tree Classifier	– Random splits lead to reduced variance. – Generally faster than Random Forest due to randomness. – Can be less prone to overfitting.	– Potentially less accurate than Random Forest. – Random splits sometimes produce suboptimal trees.
CatBoost	– Handles categorical data directly. – Less prone to overfitting with default parameters. – Built-in support for missing data. – Has an efficient implementation.	– Can be slower to train compared to other Gradient-Boosting Machine (GBM)s. – Parameter tuning can be complex for novice users.
LightGBM	– Fast training and efficient memory usage. – Supports categorical features. – Suitable for large datasets with improved accuracy. – Uses gradient-based one-side sampling.	– More sensitive to overfitting with small datasets. – Requires careful tuning for optimal performance. – Might be less intuitive than traditional GBMs.

To enhance the efficacy of our machine learning models, we engaged in a detailed process of hyperparameter tuning. This step involved adjusting key parameters such as the learning rate, number of trees, and maximum depth of the trees (Hernández-Lobato et al., 2017). The importance of fine-tuning hyperparameters cannot be overstated, as the performance of machine learning models is often significantly influenced by these parameters, a concept emphasized by Hutter et al. (2019). Our approach involved employing a random search strategy, which entailed a systematic evaluation of the model’s performance across a spectrum of hyperparameter configurations. This evaluation was conducted using a fivefold flat cross-validation process on the training data that had been set aside (Wainer and Cawley, 2021). Such a method proved invaluable in determining the most effective hyperparameter combinations for the final training of our model. In our research, we explored a diverse range of algorithms, each characterized by unique classification strategies and varying parameter values shown in Table 3.

**TABLE 3:** Parameters used during model development.

Algorithm	Critical parameters used during model development	Tested range/values
Logistic Regression	C: This parameter controls the strength of the L2 regularization. A higher value of C will lead to a more regularized model, which may be less accurate but more robust to overfitting.	(0.01, 0.03, 0.04, 0.05, 0.06, 0.08, 0.1, 0.2, 0.3, 0.4, 0.5, 0.6, 0.8, 0.9, 0.1, 0.2, 0.3, 0.4, 0.5, 0.8, 1, 1.5, 2, 2.5, 3, 3.5, 4, 5)
	Penalty: This parameter is used to specify the norm used in penalization.	l2, l1
	Class_weight: This parameter is used to specify weights associated with classes in order to address imbalances in the training set.	Balanced, none
Random Forest	n_estimators: This parameter controls the number of trees in the forest. A higher number of trees will lead to a more accurate model, but it will also take longer to train.	(100, 200, 300, 400)
	Max_depth: This parameter controls the maximum depth of each tree in the forest. A deeper tree will be able to learn more complex relationships between features, but it will also be more prone to overfitting.	(3, 5, 6, 7, 8, 9, 10, 11, 12)
Decision Tree	Max_depth: This parameter controls the maximum depth of the tree. A deeper tree will be able to learn more complex relationships between the features, but it will also be more prone to overfitting.	(3, 4, 5, 6, 7, 8, 9, 10, 11, 12, 13, 14, 15)
	Max_features: This parameter defines the number of features to consider when looking for the best split.	auto, sqrt, log2
AdaBoost	n_estimators: This parameter controls the number of base learners in the ensemble. A higher number of base learners will lead to a more accurate model, but it will also take longer to train.	(100, 200, 300, 400)
	Learning_rate: This parameter controls the learning rate of the AdaBoost algorithm. A higher learning rate will make the algorithm learn more quickly, but it may also make it more prone to overfitting.	(0.01, 0.03, 0.05, 0.08, 0.1, 0.3, 0.4, 0.5, 0.6, 0.7, 0.8, 1, 1.4, 1.5, 1.6, 1.7, 1.8, 2.0)
XGBoost	Max_depth: This parameter controls the maximum depth of each tree in the forest. A deeper tree will be able to learn more complex relationships between the features, but it will also be more prone to overfitting.	(3, 4, 5, 6, 7, 8)
	Learning_rate: This parameter controls the learning rate of the XGBoost algorithm. A higher learning rate will make the algorithm learn more quickly, but it may also make it more prone to overfitting.	(0.01, 0.03, 0.05, 0.08, 0.1, 0.2, 0.3, 0.4, 0.5, 0.7, 0.9, 1.0, 1.1, 1.3, 1.5)
Extra Tree Classifier	n_estimators: This parameter controls the number of trees in the forest. A higher number of trees will lead to a more accurate model, but it will also take longer to train.	(100, 200, 300, 400)
	Max_depth: This parameter controls the maximum depth of each tree in the forest. A deeper tree will be able to learn more complex relationships between the features, but it will also be more prone to overfitting.	(3, 4, 5, 6, 7, 8, 9, 10, 11, 12, 14)
CatBoost	Iterations: This parameter controls the number of iterations the algorithm will run.	(50, 100, 150, 200)
	Depth: This parameter controls the depth of the tree. A deeper tree can model more complex relationships, but may lead to overfitting.	(2, 3, 4, 5, 6)
	Early_stopping_rounds: This parameter stops the training early to prevent overfitting in case the model’s performance does not improve after a specified number of rounds.	(1, 2, 3, 4, 5, 6, 7, 8, 9)
	Learning_rate: This parameter determines the step size at each iteration while moving toward a minimum value for the loss function. A lower value will make the optimization more robust, but the convergence will be slower.	(0.01, 0.03, 0.05, 0.08, 0.1, 0.3, 0.5, 0.8, 1, 1.3, 1.4, 1.5, 1.6, 1.8)
LightGBM	Boosting_type: This parameter defines the type of algorithm to run. *gbdt* stands for gradient boosting Decision Tree, *goss* stands for gradient-based one-side sampling, and *darts* stands for dropouts meet multiple additive regression trees.	gbdt, goss, dart
	Learning_rate: This parameter determines the rate at which the model corrects for errors from the previous iteration. A lower learning rate can lead to a more accurate model but will take longer to train.	(0.01, 0.03, 0.05, 0.08, 0.1, 0.3, 0.5, 0.8, 1, 1.3, 1.5)
	Num_iterations: This parameter specifies the number of boosting iterations, which corresponds to the number of trees added to the model.	(50, 100, 150)
	Early_stopping_rounds: This parameter stops the training early to prevent overfitting in case the model’s performance does not improve after a specified number of rounds.	(1, 2, 3, 4)
	Max_depth: This parameter controls the maximum depth of each tree in the forest. A deeper tree will be able to learn more complex relationships between the features, but it will also be more prone to overfitting.	(2, 3, 4, 5, 7)

### 2.7 Evaluation of machine learning models

To determine the effectiveness of the models, we utilized standard evaluation metrics such as accuracy, precision, recall, and F1 score. The outcomes of these assessments were recorded and are presented in Table 4, which provides a detailed view of the models’ performance on the test data (15% of the whole dataset).

**TABLE 4:** Evaluation metrics for machine learning models.

Term	Definition	Formulation
Accuracy	The proportion of predictions that are correct.	(True positives + true negatives) /total
Precision	The proportion of positive predictions that are correct.	True positives/(true positives + false positives)
Recall	The proportion of actual positive cases that are correctly predicted.	True positives/(true positives + false negatives)
F1 score	A harmonic mean of Precision and Recall.	2 × (Precision × recall)/(precision + recall)

The area under the curve (AUC) is a metric for evaluating binary classifiers computed on the receiver operating characteristic (ROC) curve. The ROC curve shows the trade-off between the model’s true positive rate (sensitivity) and its false positive rate (1–specificity) at various threshold settings (Witten et al., 2016). Fundamentally, the AUC, whose value ranges from 0 to 1, assesses the model’s proficiency in accurately distinguishing between positive and negative instances. A score of 1 represents perfect classification accuracy, whereas a score of 0.5 indicates an accuracy level equivalent to random guessing. Additionally, the AUC metric is particularly useful in our data set thanks to its resilience against imbalanced class distributions in the dataset.

To assess the fairness of the machine learning models, we analyzed performance metrics across different demographic groups, including groups based on age, gender, and educational background. We examined the performance metrics to detect any biases in model predictions. This evaluation aimed to provide an unbiased understanding of the model’s performance variations among these subgroups.

### 2.8 Explainability of the machine learning models

Assessing the explainability of our model was achieved through various techniques including SHAP (SHapley Additive exPlanations) and feature importance (Samek et al., 2017). The feature importance method quantifies the significance of each attribute in our predictive model. We employed the permutation importance method for this purpose, which involves randomly shuffling each feature in the training set and observing the resulting variations in the model’s performance (Samek et al., 2017). Features that substantially affected the model’s accuracy were identified as the most crucial.

Additionally, we utilized the SHAP method, a model-agnostic technique for elucidating predictions made by machine learning models. This method is grounded in the Shapley values from game theory, which assesses the contribution of each player (or feature) to a collective outcome (Wang et al., 2021). In the context of machine learning, SHAP values represent the mean difference in the model’s prediction with the inclusion or exclusion of a particular feature. These values, when aggregated, provide an explanation for the model’s prediction on a specific input (Wang et al., 2021). The SHAP approach offers several advantages over other explanatory methods, notably its model-agnostic nature, consistency, and local interpretability, as noted by Doshi-Velez and Kim (2017). Moreover, SHAP values are instrumental in identifying key features that substantially influence the model’s predictive behavior.

## 3 Results

### 3.1 Statistical analysis

The statistical analyses were carried out via SPSS v22.0 with 4-way Repeated Measures ANOVA using decisions (Yes, No) as the within-subject factor, optode regions (left, mid-left, mid-right, right), and optode left–right and up–down placements as the between-subject factors.

#### 3.1.1 Model assumptions

Before conducting this 4-way Repeated Measures ANOVA, several key assumptions were checked to ensure the validity of the analysis. These assumptions included normality test, sphericity (Mauchly’s test of sphericity), homogeneity of variances (Levene’s test), multicollinearity, and absence of significant outliers. For the outputs in which sphericity was violated, adjustments to the degrees of freedom (using Greenhouse-Geisser or Huynh-Feldt corrections) were applied.

#### 3.1.2 Multivariate tests

The results of the multivariate tests indicated that the optode region was significant [*F*(3, 30) = 4.656, *p* = 0.01]. The post-hoc comparisons were performed via multiple comparison tests with Bonferroni correction. The results of pairwise comparisons demonstrate that the neural activations in the left region are significantly higher than in the right region (*p* = 0.007). There were significant decision * optode region [*F*(3, 30) = 2.942, *p* = 0.05], and decision * optode region * left–right placement interactions [*F*(3, 30) = 4.261, *p* = 0.05]. There were also marginally significant decision * optode region * up–down placement [*F*(3, 30) = 2.765, *p* = 0.059]; optode region * left–right * up–down placements [*F*(1, 32) = 3.431, *p* = 0.073]; and for decision * optode region * left–right * up–down placements interactions [*F*(3, 30) = 2.758, *p* = 0.060].

#### 3.1.3 Tests of within-subjects effects

The results of tests of within-subjects effects indicate that there was a main effect for the optode region [*F*(2.646, 84.675) = 5.392, *p* = 0.002]. The results illustrate that there are significant decision * optode region [*F*(3, 96) = 3.884, *p* = 0.011], decision * optode region * left–right placement [*F*(3, 96) = 4.255, *p* = 0.007] and decision * optode region * up–down placement interactions [*F*(3, 96) = 2.840, *p* = 0.042]. Moreover, there are marginally significant levels of interaction effects for decision * left–right * up–down placement [*F*(1, 32) = 3.431, *p* = 0.073], and optode region * left–right * up–down placement [*F*(3, 96) = 2.507, *p* = 0.064]. The post-hoc comparisons were performed via multiple comparison tests with Bonferroni correction.

### 3.2 Model performance outputs

Regarding the model development phase, the model evaluation metrics were generated and presented as tables. The performance outputs of undersampling and oversampling strategies in our model development is given in Tables 5–7. These tables present a comprehensive classification report for each predictive model under three distinct data balancing methods: the original dataset, undersampling, and oversampling.

**TABLE 5:** Classification report for the predictive models.

Model	Accuracy	Precision	Recall	F1	AUC
Logistic Regression	0.78	0.74	0.77	0.77	0.77
Random Forest	0.77	0.77	0.77	0.77	0.77
Decision Tree	0.50	0.47	0.55	0.46	0.55
Ada Boost	0.74	0.73	0.73	0.73	0.73
XGBoost	0.71	0.67	0.70	0.70	0.70
Extra Tree	**0.79**	**0.78**	**0.78**	**0.78**	**0.78**
Cat Boost	0.73	0.72	0.71	0.71	0.71
LightGBM	0.77	0.70	0.76	0.77	0.76

The bold values represent the results of the best model achieved in the corresponding framework.

**TABLE 6:** Classification report for the predictive models with undersampling.

Model	Accuracy	Precision	Recall	F1	AUC
Logistic Regression	0.77	0.73	0.75	0.76	0.75
Random Forest	0.77	0.72	0.74	0.75	0.74
Decision Tree	0.63	0.63	0.65	0.63	0.65
Ada Boost	0.71	0.67	0.70	0.70	0.70
XGBoost	0.65	0.65	0.66	0.65	0.66
Extra Tree	**0.77**	**0.76**	**0.77**	**0.77**	**0.77**
Cat Boost	0.70	0.66	0.68	0.68	0.68
LightGBM	0.77	0.73	0.76	0.77	0.76

The bold values represent the results of the best model achieved in the corresponding framework.

**TABLE 7:** Classification report for the predictive models with oversampling.

Model	Accuracy	Precision	Recall	F1	AUC
Logistic Regression	0.77	0.73	0.75	0.76	0.75
Random Forest	0.79	0.75	0.77	0.77	0.77
Decision Tree	0.75	0.67	0.73	0.74	0.73
Ada Boost	0.70	0.68	0.70	0.70	0.70
XGBoost	0.74	0.74	0.74	0.74	0.74
Extra Tree	**0.75**	**0.74**	**0.73**	**0.73**	**0.73**
Cat Boost	**0.80**	**0.78**	**0.79**	**0.79**	**0.79**
LightGBM	0.77	0.71	0.76	0.76	0.76

The bold values represent the results of the best model achieved in the corresponding framework.

In the original dataset (Table 5), the Extra Tree model demonstrated the highest overall effectiveness, with an accuracy of 0.79 and an AUC of 0.78. Meanwhile, the Decision Tree model was the least effective across all metrics. Upon the application of undersampling (Table 6), we observed a general decrease in performance metrics for most models. Conversely, the implementation of oversampling (Table 7) led to an overall improvement in model performance. Notably, the CatBoost model excelled in this scenario, achieving the highest accuracy and AUC of 0.80 and 0.79, respectively.

### 3.3 Model explainability

**FIGURE 2:**
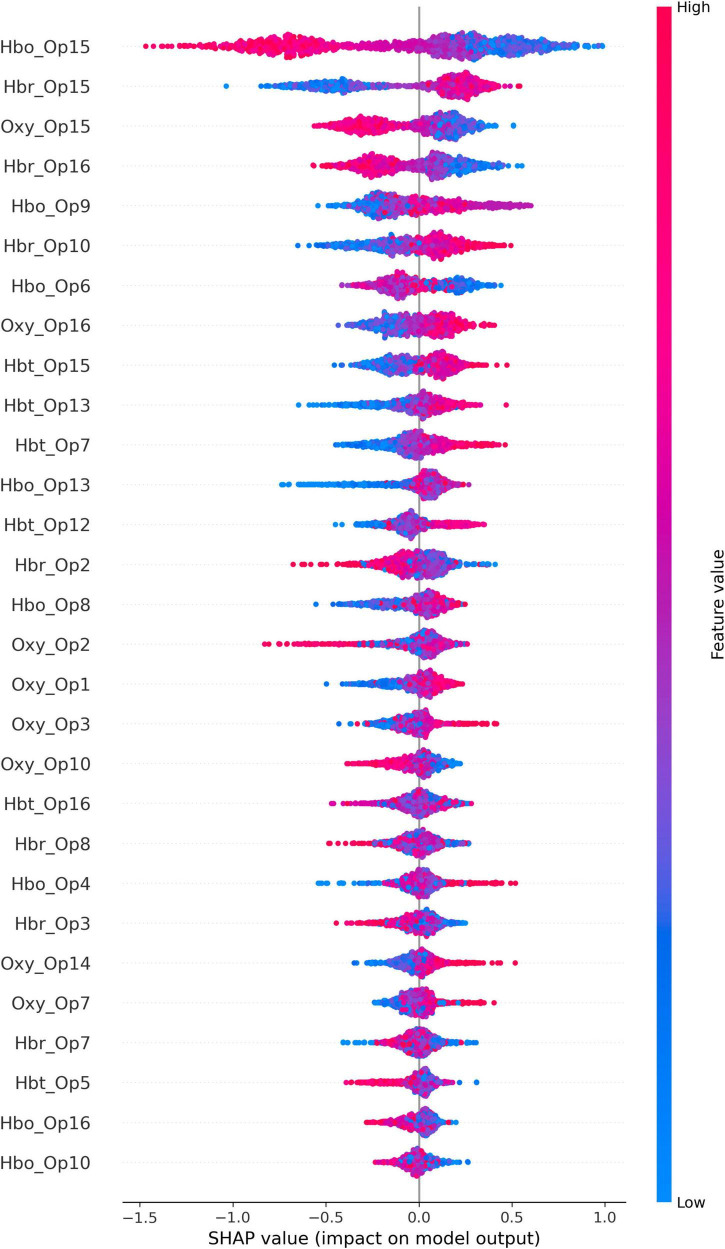
SHAP output of the CatBoost model with oversampling.

The SHAP analysis output (presented in Figure 2) illustrates how the activation patterns of different brain regions influenced model prediction, helping to reveal the importance and impact of each region on the decision-making process. Figure 2 illustrates the critical input parameters that have acquired importance during the model development phase which is generated using the test dataset.

According to the fNIRS recordings, the HbR measurements of optode 3 (corresponding to the left dlPFC), the HbT measurements of optode 5, and HbO measurements of optode 6 (corresponding to the left dmPFC), the HbR measurements of optode 8 [corresponding to the left frontopolar cortex (FPC)], the Oxy and HbO measurements of optode 10 (corresponding to the right FPC), and the HbO and Oxy measurements of optode 15, and HbR measurements of optode 16 (corresponding to the right dlPFC) correlated to a negative impact on the credit decision of participants. On the other hand, the Oxy measurements of optodes 1 and 3, and the HbO measurements of optode 4 (corresponding to the left dlPFC), the HbT measurements of optode 7 and the HbO measurements of optode 8 (corresponding to the left FPC), the HbO measurements of optode 9 (corresponding to the right FPC), the HbT measurements of optode 12 (corresponding to the right dmPFC), the HbO and HbT measurements of optode 13, Oxy measurements of optode 14, HbR and HbT measurements of optode 15, and Oxy measurements of optode 16 (all of which correspond to the right dlPFC) correlated to a positive impact on the credit decision of participants.

In Table 8, each feature is associated with an importance score, which quantifies the contribution of that feature to decision-making process of the CatBoost model with oversampling. These importance scores are computed based on the model’s internal calculations, such as how often each feature is used to split the data and make predictions. It was observed that the feature importance outputs were compatible with the SHAP analysis results. The obtained findings support our initial hypothesis for the activation of vmPFC, dmPFC, and dlPFC regions for this experimental task, since the related optodes acquired importance within the SHAP outputs.

**TABLE 8:** Feature importance results of the CatBoost model with oversampling.

Feature	Importance	Feature	Importance
Hbo_Op15	8.23	Oxy_Op1	3.06
Hbo_Op9	5.65	Hbo_Op13	3.06
Hbr_Op15	5.31	Hbo_Op4	3.05
Hbr_Op10	5.16	Oxy_Op16	3.05
Hbr_Op16	4.04	Hbo_Op8	3.04
Hbr_Op8	4.01	Hbt_Op16	3.03
Oxy_Op2	3.90	Oxy_Op3	2.92
Hbr_Op2	3.63	Oxy_Op10	2.79
Hbo_Op6	3.63	Hbr_Op7	2.71
Hbt_Op13	3.54	Oxy_Op14	2.43
Oxy_Op15	3.38	Hbo_Op10	2.05
Hbt_Op7	3.30	Oxy_Op7	2.02
Hbt_Op12	3.25	Hbt_Op5	1.95
Hbr_Op3	3.16	Hbo_Op16	1.52
Hbt_Op15	3.13		

### 3.4 Evaluation of model fairness

The fairness of the developed best model is evaluated by the assessment of the model’s performance on different subgroups of the population.

For age groups, the model showed higher accuracy, precision, recall, and F1 scores for the younger group (25–33 years), with an overall accuracy of 0.86 (Table 9). In contrast, the performance for the 34–43 age group denoted a notable decrease in precision, recall, and F1 scores for positive cases, resulting in a lower overall accuracy of 0.75 (Table 9).

**TABLE 9:** Evaluation metrics for age groups.

	Precision	Recall	F1 score	Support
**Age group 1 (25–33)**				
0 (negative)	0.84	0.90	0.87	48
1 (positive)	0.88	0.82	0.85	45
Accuracy			0.86	93
Macro average	0.86	0.86	0.86	93
Weighted average	0.86	0.86	0.86	93
**Age group 2 (34–43)**				
0 (negative)	0.79	0.82	0.80	65
1 (positive)	0.66	0.62	0.64	37
Accuracy			0.75	102
Macro average	0.72	0.72	0.72	102
Weighted average	0.74	0.75	0.74	102

Gender-wise analysis revealed a visible disparity. The performance metrics for females were exceptionally high with an accuracy of 0.98, indicating almost perfect classification (Table 10). However, the metrics for males were lower, with an accuracy of only 0.63 (Table 10).

**TABLE 10:** Evaluative metrics for gender groups.

	Precision	Recall	F1 score	Support
**Male**				
0 (negative)	0.69	0.72	0.70	61
1 (positive)	0.54	0.50	0.52	40
Accuracy			0.63	101
Macro average	0.61	0.61	0.61	101
Weighted average	0.63	0.63	0.63	101
**Female**				
0 (negative)	0.96	1.00	0.98	52
1 (positive)	1.00	0.95	0.98	42
Accuracy			0.98	94
Macro average	0.98	0.98	0.98	94
Weighted average	0.98	0.98	0.98	94

Furthermore, when evaluating the model’s fairness in terms of educational background, we observed a similar performance for both high school graduates and bachelor’s degree holders, with accuracies of 0.81 and 0.80, respectively (Table 11).

**TABLE 11:** Evaluative metrics for educational background groups.

	Precision	Recall	F1 score	Support
**High school graduates**				
0 (negative)	0.79	0.89	0.84	38
1 (positive)	0.84	0.70	0.76	30
Accuracy			0.81	68
Macro average	0.82	0.80	0.80	68
Weighted average	0.81	0.81	0.81	68
**Bachelor’s degree holders**				
0 (negative)	0.83	0.83	0.83	75
1 (positive)	0.75	0.75	0.75	52
Accuracy			0.80	127
Macro average	0.79	0.79	0.79	127
Weighted average	0.80	0.80	0.80	127

## 4 Discussion

Financial decisions constitute some of the most important decisions individuals make and can significantly influence the course of their life. Thus, understanding every aspect of financial preferences is crucial. The behavior of getting a loan should be carefully considered by the recipient. Even though this evaluation postulates that economic wellbeing exerts a positive influence on secondary rewards, a prudent assessment of the feasibility of timely repayment within the present circumstances is equally imperative. Thus, one can think of it as a struggle between potential gains balanced against potential losses. The current study aimed to reveal the regions of the PFC that hold a potentially predictive role in individuals’ financial preferences when evaluating credit decisions during the recording of hemodynamic responses of participants. The findings obtained from the statistical analyses confirm that the hemodynamic activity tends to change with respect to the credit decisions given this experimental design.

The study integrated fNIRS data with different machine learning algorithms, namely Logistic Regression, Random Forest, Decision Tree, AdaBoost, XGBoost, Extra Tree Classifier, CatBoost, and LightGBM to predict the participants’ credit decisions based on prefrontal cortex (PFC) activation patterns up to 80% accuracy in oversampling condition. In addition to this, behavioral findings obtained indicated that the participants tended to respond more frequently to negative buttons (62%) than to positive buttons (38%). The neuroscientific findings revealed distinct PFC regions associated with credit behaviors, including the dlPFC, the OFC, the vmPFC and the dmPFC.

### 4.1 Evaluation of model performance outputs

This study provides valuable insights into the complex relationship between data balancing techniques and model performance for imbalanced datasets. By carefully choosing and implementing appropriate balancing methods, researchers can significantly enhance the effectiveness of their models, leading to more accurate and reliable predictions. However, it is crucial to maintain a balanced perspective, considering both the potential benefits and risks associated with different approaches. Through continued research and exploration, we can develop robust and ethical AI models capable of delivering superior performance across diverse data scenarios.

Three distinct data balancing techniques were employed: original dataset, undersampling, and oversampling. The performance of each model under these different scenarios is analyzed and discussed. The findings highlight the importance of data balancing techniques in optimizing model performance for imbalanced datasets. While the Extra Tree model performed well in the original unbalanced dataset, oversampling further enhanced the performance of the CatBoost model. This demonstrates that different models respond differently to data balancing techniques, and the optimal approach may require careful evaluation and experimentation. However, it is important to acknowledge potential drawbacks of oversampling. Oversampling can introduce artificial data points, potentially leading to model overfitting and reduced generalizability. Therefore, careful validation and evaluation are crucial to ensure that the benefits of oversampling outweigh its potential risks. In line with these, the Decision Tree model exhibited an improvement, particularly in precision and recall, suggesting that this model is more sensitive to class imbalance. Notably, the CatBoost model excelled in this scenario, achieving the highest Accuracy and AUC of 0.80 and 0.79, respectively. This indicates a potential affinity of this model to the oversampling technique.

### 4.2 Analysis of the model’s fairness

The fairness of a developed model by analyzing its performance across various demographic subgroups were investigated. The findings indicate age and gender biases in the model’s predictions, while the results for educational background reveal more moderate performance biases.

#### 4.2.1 Age-related bias

The model exhibits a distinct performance difference between the younger (25–33 years) and older (34–43 years) age groups. The younger group benefits from higher accuracy and precision, recall, and F1 scores, indicating a potential bias favoring their predictions (Table 9). Even though this difference does not indicate severe bias, it might be overcome with larger sample size.

#### 4.2.2 Gender-related bias

The observed gender disparity is particularly concerning. The lower accuracy for males (0.63) compared to females (0.98) indicates a pronounced bias that systematically disadvantages male individuals (Table 10). This raises ethical concerns about the model’s potential discriminatory implications and necessitates immediate mitigation strategies to ensure fairness and inclusivity. Even though gender-based differences have been empirically proven in the relevant academic literature (Cazzell et al., 2012; Duan et al., 2021; Nissen and Krampe, 2021; Aryadoust et al., 2022), for the current study the difference might be related to the motivation level of the participants, which could have resulted in a gender-based difference.

#### 4.2.3 Educational background bias

The model’s performance for different educational backgrounds (Table 11) do not exhibit a distinct difference where both high school graduates and bachelor’s degree holders achieve similar accuracies (0.81 and 0.80, respectively).

### 4.3 Model explainability

The models we developed based on fNIRS measurements yield significant insights into participants’ reactions to loan offers. However, it is important to recognize that fNIRS signals do not always correlate directly with specific brain regions. This variability in signal interpretation across different studies is attributed to the nature of fNIRS technology, which tracks changes in blood oxygenation levels. These levels are influenced by multiple factors, including blood flow, metabolic processes, and brain activity. Despite these inherent limitations, fNIRS stands out as a reliable instrument for examining brain activity, especially in scenarios where alternative methods like fMRI may not be practical or available. This versatility underscores the utility of fNIRS in neuroscientific research, particularly in applied settings outside traditional laboratory environments. The obtained findings of this research underscore the critical role of the PFC in shaping financial preferences and decisions. Notably, distinct regions within the PFC exhibited varying patterns of activation that correlated with participants’ credit decisions. The outputs from the developed best model indicate that our initial hypothesis is supported by the feature importance and SHAP outputs by which specific PFC regions including vmPFC, dmPFC, and dlPFC tend to have roles during the credit decision-making process.

#### 4.3.1 dlPFC

The dlPFC has an indispensable role in strategic decision-making (Soutschek et al., 2015). Moreover, the activity of the left dlPFC regulates impulsivity and strategic behavior; in particular, increased activity in the left dlPFC causes an important decrease in risk-taking. However, decreased activity in the left dlPFC suppresses strategic behavior but does not trigger risk-taking (Fecteau et al., 2007; Steinbeis et al., 2012). The current findings showed that the activity pattern of the left dlPFC (optodes 1-Oxy, 3-Oxy, and 4-HbO) correlated with impact tendency on credit preference: increased activity in the left dlPFC correlated with a positive impact on credit behaviors, while, increased activity in the left dlPFC (optode 2-Oxy) correlated with a negative impact on credit decisions. These findings suggest that the activation observed in the left dlPFC is a potential indicator for either positive or negative behavioral outcomes within individuals, in line with the relevant scholarly literature. It has been shown that the right anterior polar cortex has a significant impact on purchase decisions (Mitsuda et al., 2012; Çakır et al., 2018). Increased levels of neural activity in the right dlPFC are associated with maintaining alertness and verbal reasoning, and decreased activity is associated with risk-taking in the context of loss, especially when protection is a concern (Mannarelli et al., 2015; Huang et al., 2017; Obeso et al., 2021). These findings showed that an increase in neural activity in the right dlPFC is correlated with positive credit decisions. However, the opposing nature of activity in this region (optode 15), in contrast to other areas, may be related to the right dlPFC accelerating the rate of adaptation to visual rotation by enabling participants to explicitly explore the movement space by more actively changing their aiming direction (Song et al., 2020). These findings are in line with the literature mentioned above and suggest that the activation patterns of the right dlPFC are a promising prediction metric for risky decisions (Yang et al., 2017).

#### 4.3.2 OFC

The frontopolar cortex is closely associated with the OFC and vmPFC regions (Koechlin, 2011; Nashiro et al., 2013; Çakır et al., 2018; Bak et al., 2022). The OFC, a subregion of the prefrontal cortex, has demonstrated consistent activation across multiple investigations in domains such as commercial tactics, consumer preferences, product preference discernment, risk appraisal, financial investment choice, and pricing evaluations (Kim et al., 2016). The anterior part of the OFC is associated with the representation of fundamental reinforcers such as taste, and is also involved in updating associations to emotional stimuli, whereas the posterior part is implicated in processing more intricate or abstract reinforcements, including financial gains and losses (Kringelbach, 2005; Sakaki et al., 2011). It has also been demonstrated that good-based decisions take place in the OFC and thus regulate goal-directed actions; consequently, the OFC has deactivation in low-profit margin (Gremel and Costa, 2013; Padoa-Schioppa and Conen, 2017; Wanniarachchi et al., 2021). The left OFC (optode 8-HbO) correlates with increased activity in positive credit decisions. On the other hand, the right OFC (optode 10-Oxy and -HbO) shows increased hemodynamic activity during negative credit decisions. In line with these findings, the discrete correlation of both left and right OFC with credit decisions is a promising outcome for the predictive modeling of people’s decisions.

#### 4.3.3 vmPFC

Another prefrontal region related to the FPC is the vmPFC. The findings of this study indicate that bilateral increased vmPFC (optodes 7-Oxy and 9-HbO) activity is associated with positive credit decisions. It has been shown that the bilateral vmPFC activity increases during preference judgments (Paulus and Frank, 2003) or normative judgments (Baumgartner et al., 2011). Also, it has been shown that the increase in activation of the vmPFC may be a process of integrating previous emotional experiences with the brand into the ongoing decision process (Deppe et al., 2005). Hence, the bilateral increased activation of the vmPFC correlates with positive credit decision; this is consistent with the current literature and suggests that the vmPFC (predominantly the left vmPFC) is an important region for predicting behavioral outcomes of individuals as its involvement in the interaction of brand information and uncertainty information (Plassmann et al., 2008).

#### 4.3.4 dmPFC

The dmPFC contributes to strategic control in complex decision-making processes (Venkatraman et al., 2012). However, together with the vmPFC, it takes a role in processing social and economic information. The vmPFC and the dmPFC provide divergent contributions to the processing of subjective reward valuations and normative reward valuations. The dmPFC only processes the value of rewards when making normative choices, whereas value encoding takes place in the vmPFC (Apps and Ramnani, 2017). Decreased activation in the dmPFC results in the suppression of normative behavior, and in strategic control, while an increase in vmPFC activity is associated with reward values. There is no finding that supports the relation between the dmPFC and the vmPFC, yet the increased activity in both left and right vmPFC is associated with positive credit decisions. In addition, the increased levels of hemodynamic activity in the left dmPFC (optodes 5-HbT and 6-HbO) is associated with negative credit decisions; this indicates that the left dmPFC might play a role in people being risk-averse in their loan-taking behavior. Despite that, the increased hemodynamic activity in the right dmPFC (optode 12-HbT) correlates with positive credit decisions. In line with the current findings, these two regions separately appear to be possible indicators of credit decision-making processes. Their effects on behavioral outcomes shall be studied with more specific and detailed experimental designs, with the prediction that the unique activation balance of these two regions is likely to provide potential evidence for predicting people’s decisions.

### 4.4 Limitations

The limitations of the study are primarily associated with the inherent constraints of functional near-infrared spectroscopy (fNIRS) during the data collection and interpreting the results. First, specific to this fNIRS model, it is limited to data collection from the prefrontal cortex and the lateral regions might cause problems during the data collection due to the forehead. Also, the experimental design does not allow to pinpoint the exact moment of decision-making, implying that the results most likely represent offer evaluation. Secondly, despite the compelling findings, it is crucial to acknowledge that fNIRS, as a method, has limitations that can affect the interpretation of the data (Arenth et al., 2007; Ferrari and Quaresima, 2012; Boas et al., 2014; Rupawala et al., 2018; Hussain et al., 2023). These include susceptibility to noise and artifacts present in the fNIRS data and to address these limitations, some researchers have employed inverse problem solutions. These solutions are mathematical algorithms that utilize knowledge of light propagation physics within the brain to estimate the origin of fNIRS signals (Tremblay et al., 2018; Condy et al., 2021). However, it is important to note that these inverse problem solutions also have their own limitations, including vulnerability to the noise and artifacts inherent in fNIRS data (Kirilina et al., 2012; Hussain et al., 2023).

In addition to the limitations associated with the fNIRS methodology, the study’s design, primarily based on laboratory experiments, presents another set of constraints. Laboratory experiments, while controlled and replicable, may not always accurately represent real-world scenarios. The artificial environment of a laboratory can influence participants’ behavior, potentially leading to results that do not fully translate to natural settings. This limitation is important to consider, as the study’s findings might vary if conducted in a more realistic context outside the laboratory.

### 4.5 Future prospects

This research highlights how data balancing techniques impact model performance in imbalanced datasets. By selecting and applying suitable balancing methods, researchers can improve model effectiveness, yielding more precise and dependable predictions. It might be of interest to investigate the factors contributing to the performance differences observed across models under various data balancing techniques. Another future research might focus on exploring the effectiveness of alternative data balancing methods, such as SMOTE or ADASYN, against the approaches employed in this study as diving deeper into the impact of oversampling on model generalizability and exploring methods to mitigate potential overfitting risks. Further research might aim to increase the number of participants while developing ensemble models that leverage the strengths of individual models and capitalize on the benefits of both undersampling and oversampling strategies.

Despite the fact that the developed model demonstrates acceptable overall performance, the identified biases raise critical concerns about its fairness and ethical implications. Addressing these issues through careful analysis, proactive mitigation strategies, and ongoing research is crucial to ensure responsible and inclusive development and deployment of AI systems. It might be of research interest to investigate the specific features or data points responsible for the observed biases, explore alternative modeling techniques or algorithms that are less susceptible to bias, develop standardized benchmarks and evaluation metrics for assessing fairness in machine learning models. However, it is crucial to maintain a balanced perspective, considering both the potential benefits and risks associated with different approaches. Through continued research and exploration, it is possible for the researchers to develop robust and ethical AI models capable of delivering superior performance across diverse data scenarios.

### 4.6 Conclusive remarks

Overall, the current investigation bridges the gap between neuroscience and finance, offering novel insights into the neural mechanisms within the prefrontal cortex that underlie consumer credit evaluations. By unraveling the neural signatures of financial decision-making, this research opens avenues for the development of innovative strategies that leverage neuroscientific knowledge to inform marketing practices and optimize financial decision support systems. As the fields of neuromarketing, neurofinance and behavioral economics continue to evolve, this study demonstrates the potential of interdisciplinary research in reshaping our understanding of human behavior in financial contexts. The contributions of this study in predicting credit decisions based on neural activation patterns underscores the potential of fNIRS coupled with machine learning algorithms in deciphering intricate cognitive processes underlying financial behaviors. Such predictive modeling can provide valuable insights for both researchers and financial institutions, enabling a deeper understanding of consumer behavior and facilitating tailored financial services.

## Data availability statement

The raw data supporting the conclusions of this article will be made available by the authors, without undue reservation.

## Ethics statement

The studies involving humans were approved by the MEF University Ethics Committee (E-47749665-050.01.04/275). The studies were conducted in accordance with the local legislation and institutional requirements. The participants provided their written informed consent to participate in this study.

## Author contributions

TÇ: Conceptualization, Funding acquisition, Investigation, Methodology, Project administration, Writing – original draft, Writing – review & editing. SST: Conceptualization, Formal Analysis, Investigation, Methodology, Project administration, Writing – original draft. YG: Conceptualization, Investigation, Project administration, Resources, Supervision, Writing – original draft. AS: Data curation, Methodology, Validation, Visualization, Writing – original draft. SE: Conceptualization, Data curation, Investigation, Supervision, Writing – original draft. GF: Data curation, Methodology, Validation, Visualization, Writing – review & editing. ET: Conceptualization, Resources, Validation, Writing – review & editing.

## Footnotes

1
www.fnirdevices.com
